# Group therapy on in utero colonization: seeking common truths and a way forward

**DOI:** 10.1186/s40168-020-00968-w

**Published:** 2021-01-12

**Authors:** Rachel B. Silverstein, Indira U. Mysorekar

**Affiliations:** 1 Department of Obstetrics and Gynecology, Washington University in St. Louis School of Medicine, St. Louis, MO 63110 USA; 2 Department of Pathology and Immunology, Washington University in St. Louis School of Medicine, St. Louis, MO 63110 USA

**Keywords:** Decidua, Pregnancy, Placenta, Extravillous trophoblasts, Microbiome, Low biomass microbial communities, Ralstonia, Micrococcus, Kitome

## Abstract

The human microbiome refers to the genetic composition of microorganisms in a particular location in the human body. Emerging evidence over the past many years suggests that the microbiome constitute drivers of human fate almost at par with our genome and epigenome. It is now well accepted after decades of disbelief that a broad understanding of human development, health, physiology, and disease requires understanding of the microbiome along with the genome and epigenome. We are learning daily of the interdependent relationships between microbiome/microbiota and immune responses, mood, cancer progression, response to therapies, aging, obesity, antibiotic usage, and overusage and much more. The next frontier in microbiome field is understanding when does this influence begin? Does the human microbiome initiate at the time of birth or are developing human fetuses already primed with microbes and their products in utero. In this commentary, we reflect on evidence gathered thus far on this question and identify the unknown common truths. We present a way forward to continue understanding our microbial colleagues and our interwoven fates.

## Bacteria R Us

**Fig. 1:**
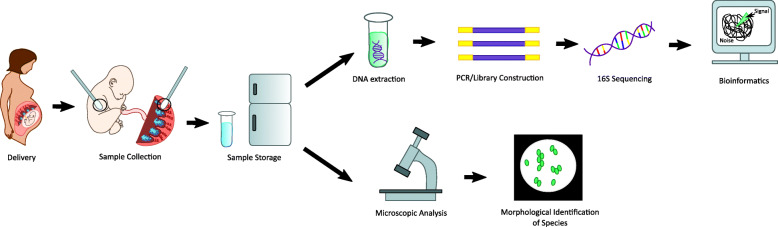
Molecular and cellular visualization methods for microbiome analysis at maternal fetal interface and in fetal gut/meconium

The human body is home to a variety of microbes, including bacteria, archaea, fungi, microbial eukaryotes, and viruses/phages. Our bacterial friends are in an overall 1:1 stoichiometric relationship with human cells. Thus, understanding how we have co-evolved and how we affect each other remains of the greatest importance. The microbes around us have the power to modulate not only our external environment, such as the soil and food we consume, but also have a profound impact on the internal environment of the human beings they inhabit. It comes as no surprise therefore that the state of pregnancy, with its accompanying metabolic and immunological changes, alters the microbiota at a variety of body sites including the gut, oral mucosa, vaginal mucosa. Several studies have even linked microbial community alterations to being affected by maternal conditions such as diabetes [1, 2], excess gestational weight gain [3], or eczema [4], with others suggesting links between the microbiota and clinical outcomes such as low birth weight [5] and preterm birth [6–8]. Advancements in technologies and methodologies to identify the components of the microbiota, including culture-independent methods, next-generation sequencing and bioinformatics have begun to provide a clearer picture of the types and niches inhabited by microbes and the types of microbial communities within us [9, 10]. Despite humans being half bacteria and half human in terms of the number of cells, bacteria are unevenly distributed across our body sites with high density in our gut, mouth, skin, nose, and vagina. Next-generation sequencing approaches have evolved to be highly sensitive, which has allowed for the identification of other sites such as the urine, uterus, penile urethra, lower airway, within tumors, and the maternal-fetal interface/placenta as harboring low biomass microbial communities [11] (Fig. 1).

## Group therapy: relationships are all about patterns—therapy is about analyzing the patterns together

In this commentary, we focus on two of these body sites: the maternal-fetal interface, which includes the maternal decidua and the placenta, and the fetus itself, particularly the fetal gut. The maternal-fetal interface is made up of the maternal decidua and fetally derived placenta. The placenta comprises fetal cytotrophoblast cells, which follow villous and extravillous pathways. In the villous pathway, mononuclear cytotrophoblasts fuse, creating multinucleated syncytiotrophoblasts that establish villi surrounded by maternal blood and aid in the provision of nutrients and gas exchange with fetal cells. Cytotrophoblasts also follow the extravillous pathway and differentiate into interstitial and endovascular extravillous trophoblast cells, which remodel the spiral arteries and invade into the maternal decidua to be surrounded by a large population of maternal immune cells including decidual Natural Killer cells, macrophages, T cells, and dendritic cells [12]. The developing fetal gut is divided into three segments: the foregut (esophagus/duodenum/liver and gallbladder), midgut (lower duodenum/ileum/ascending colon), and hindgut (descending colon, rectum, anal canal).

A number of studies over the past decade have hammered on whether this interface contains any bacteria or a low-biomass community. Or put more bluntly, there is a ‘controversy in the field’ resulting in two rather strong stances: that the womb is a sterile niche and any microbial signals must be contamination or that there is a bona fide microbiome at this interface. Unless otherwise noted, the terms microbes or microbiota focus on the bacterial component. We discuss a number of these studies below. In addition, there has been considerable discussion with the broader community and in the media about this topic [13, 14]. We highlight studies that present morphological evidence of microbes in the placenta and fetal gut. Given the distinct landscape differences between placental villi and maternal decidua, and between the fetal fore and hindgut, it further remains a key imperative to identify which location is being analyzed for microbial presence. The precise location of these microbes is critical to evaluate because the location can inform biological significance.

We posit that all of these studies are important as they are investigating whether (1) in utero development is affected by microbes within us or vice versa and (2) this effect commences in utero or at birth. We further posit that this meta-analysis is akin to *group therapy*, wherein a holistic look at the evidence presented from ‘both sides’ actually provides support for a small but distinct overlap between the two seemingly disparate stances that are being uncovered by next-generation sequencing methods, imaging/morphological evidence, and meta-analyses. Furthermore, the location within the maternal-fetal interface could influence the conclusion. We suggest these overlaps or common truth signal a way forward, and that a more thorough comparison of cases and controls and more studies making use of techniques (culture, FISH, histology) other than sequencing are needed to fully address the issue of contamination.

## Group therapy: let us talk about the differences

Overall, studies using 16S rRNA gene amplicon sequencing support the idea that the noise is too high to draw consistent conclusions about a ‘microbiome’ in the villous placenta. Research supporting the sterile womb hypothesis posits that data suggesting the presence of microbial communities in the placenta are actually due to contamination of placental samples during delivery or during the processing of bacterial DNA [15, 16]. For example, using 16S rRNA sequencing, De Goffau and colleagues identified several sources of contamination, including labor and delivery and sample processing that are associated with different bacterial signals and concluded that they find no clear evidence of a placental microbiome. Of note, the samples were exclusive of the fetal placental villi and did not include the maternal decidua. They identified some samples with low biomass bacterial signal, but concluded that the source of this signal was due to contamination. Similarly, Sterpu et al. found no significant differences between placental samples and controls based on 16S data, with the majority of their samples being flagged as contaminants by decontamination software [16]. However, they found that the median gene counts for bacterial signal were highest for the maternal side of the placenta regardless of the mode of delivery and indeed detected sporadic bacteria. Olomu et al. and Theis et al. also used 16S rRNA gene amplicon sequencing of placental parenchyma (villi) with positive and negative controls and did not include maternal decidua in their analyses [17, 18]. They found no significant differences in either total biomass or microbial composition when comparing placental samples and negative controls. Kuperman et al. also did not identify significant bacterial signals in the placental villi using 16S rRNA gene amplicon sequencing, but when they used other methods such as Gram stain, immunohistochemistry for bacteria, and electron microscopy, they found evidence of bacteria [19]. Parnell et al. [20] utilized multiple variable regions of the 16S gene to analyze microbial profiles at the maternal decidua and placental villi and found that villous regions did not harbor significant bacterial signals. However, they found significant bacterial gene counts in the decidua and specifically localized particular species of bacteria in this space. Bushman and colleagues did not identify any significant differences in gene copy numbers between placental villous samples and negative controls [21]. A follow-up study by the same group found little difference in the total amount of bacterial DNA detected when comparing placental samples and negative controls, or when comparing term and preterm samples [14]. However, they noted increased bacterial signals in samples which included maternal decidua and identified bacteria similar to Parnell et al. in this compartment but considered it a contaminant. Re-analysis of the two studies however noted the presence of unique bacterial DNA signatures in placental samples but not in controls, arguing that all signals could not be ascribed to contamination [22].

Research arguing for the presence of a low biomass placental microbiome argues that bacteria are able to survive and grow in placental tissues. For example, Leon et al. [23] analyzed placental samples from term and preterm births using 16S rRNA gene amplicon sequencing but observed a significant shift in the composition and abundance of bacterial species detected once contaminants and low reads were filtered out. Aagaard and colleagues [24] used 16S rRNA gene amplicon sequencing and WGS sequencing on placental samples and found bacterial sequences in the placenta. However, being the first large genomic analysis of microbes in the placenta, the authors were cognizant of the low biomass of microbiota as a significant limitation to their study. This study was followed-up by Antony et al. [4] who analyzed placental samples from term and preterm births and found differences in microbial composition in women with excess gestational weight gain (GWG) compared to those without. Importantly, they identified differences in the prevalence of different metabolic pathways in women with or without excess GWG. This opened the field to a number of studies including Bassols et al. [1] who took placental samples from patients with and without gestational diabetes (GDM) and analyzed them using 16S rRNA gene amplicon sequencing to determine that GDM status potentially altered microbial community membership at the maternal-fetal interface. Gomez-Arango and colleagues [25] built on these studies and took oral, gut, and placental samples from the same overweight or obese pregnant women and analyzed their microbial composition using 16S rRNA gene amplicon sequencing. They found that at the phyla level, placental samples shared microbes with both oral and gut microbiomes, suggesting both the oral and gut microbiomes as potential sources for seeding the placental microbiome. At lower taxonomic levels, the placental microbiome showed greater similarity to the oral microbiome; however, the placental microbiome was determined to have a unique composition. This study also compared samples to negative controls to address the issue of contamination and found that samples yielded more sequences and exhibited distinct clustering, providing further evidence that the bacteria detected were not contaminants.

Thus, overall, the studies highlighted above all identified significant sources of contamination and considerable noise in the analysis of placental villous parenchyma, but some noted signals above the noise. Notably, the areas where signals were noted included the maternal decidua, suggesting that the decidua may indeed harbor microbes.

## Group therapy: what about the kids?

In the midst of the discussion of colonization at the maternal-fetal interface, data emerged on the impact on the fetus itself: the fetal gut and meconium, which is the first stool of an infant and is composed of materials ingested in utero. For example, Hu et al. highlighted possible microbial presence in the meconium samples and controls using 16S rRNA gene amplicon sequencing [2]. In this study, the microbial composition of the meconium was similar to that of the adult gut microbiome, supporting the study’s hypothesis that microbial colonization of the developing gut begins in utero. A few years later, Collado and co-workers [26] looked at the microbial composition of samples of maternal feces, placenta, amniotic fluid, colostrum, meconium, and infant feces using 16S rRNA gene amplicon sequencing and found some similarities between the microbiomes of the placenta/amniotic fluid and the meconium. These results suggest that colonization of the fetal gut may begin in utero based on contact with the placenta and/or amniotic fluid. Further support for this hypothesis came from a study where meconium samples as well as fecal samples from pregnant women and infants during their first 7 months of life were analyzed using 16S rRNA gene amplicon sequencing. It was found that the meconium microbiota was similar to infant fecal samples but distinct from adult samples, as well as the vaginal and skin microbiota that could be potential sources of contamination [4]. Studies from Stinson et al. [27], Mshvildadze et al. [28], He et al. [29], and others [2, 26, 30, 31] suggest that most species found in the infant’s gut could also be found in the amniotic fluid possibly due to seeding from swallowed amniotic fluid or the placental interface. Thus, given the phyla-specific similarities of microbes identified in the placenta, amniotic fluid, and infant meconium, it has been proposed that the developing neonate acquires part of its complement of microbiomes from the placenta in utero [26]. Recent work from Rackaityte et al. [32] analyzed intestines from second-trimester fetuses for the presence of bacteria using scanning electron microscopy and 16S rRNA gene amplicon sequencing. Based on 16S rRNA gene amplicon sequencing, the authors found a number of taxa enriched in the meconium samples compared to negative controls. They further identified the presence of *Micrococcus luteus* and suggested that bacteria are present in the fetal intestine in utero and that they alter fetal mucosal immunity, specifically memory T cells in the lamina propria. However, de Goffau and colleagues reanalyzed these data and suggested that the data might be influenced by batch effects, as they found differences in the two batched analyses. Based on their reanalysis, the authors suggest Micrococcus as a potential contaminant because it was present in batch 1 but absent in batch 2 (in this issue of *Microbiome*). Rackaityte and coworkers had the opportunity to respond and critique the reanalysis of their data. They cited use of low sequence reads, which inflate false negatives, and for relying on Principal Component Analysis (PCA), which applies poorly to 16S rRNA gene amplicon sequencing of low burden bacterial communities. They argue that these critiques did a poor job of explaining their variation as being due to batch effects. They also refute potential batch effects by pointing out that their samples were not processed in batches, and enrichment of *Micrococcaceae* was found in both ‘batches.’ This round of metaanalyses underscores how scrutiny of two stances can provide oversight on both the validity and limitations of methodologies used and conclusions reached*.* Overall, it appears clear that batch effects notwithstanding, bacterial cocci are evident in the fetal gut.

## Group therapy: who is in the house?

A spate of studies has taken a targeted visualization approach to identify possible bacteria at the maternal-fetal interface and the fetal gut. The aforementioned work from Rackaityte et al. [30] further analyzed fetal gut samples from second-trimester fetuses for the presence of bacteria using scanning electron microscopy and found cells that appeared to be bacterial cocci in pockets of the meconium. Although De Goffau et al. critiqued this observation by pointing out that the structures identified appeared much larger than *Micrococcaceae*, and no species-specific FISH studies were performed to confirm the identity of the structures, it is not in doubt that there are bacterial cocci present in the fetal gut. Bacteria have been identified in the placental tissue using traditional culture-based techniques [32]. Steel et al. in 2005 [33] probed fetal membranes from term placental samples using fluorescent in situ hybridization (FISH) targeted to 16S rRNA. FISH revealed fluorescence that suggests the presence of bacteria in the majority of samples from term placentas, including those delivered via cesarean section. This paper is notable as one of the first studies to suggest that bacteria are present in healthy, term pregnancies. Stout and co-workers in 2013 [34] specifically collected maternal decidual and placental villous samples and provide extensive morphological evidence of bacteria present in over a third of all placentas using Hema 3 Geimsa, Gram, and Brown and Hopps stains. These findings indicate that whole bacteria could be present in the maternal decidua in normal term placentas and that the presence of bacteria may not necessarily be associated with adverse pregnancy outcomes. Cao and Mysorekar [35] showed that bacteria reside intracellularly within HLA-G+ extravilllous trophoblasts (EVTs) and that bacteria are able to colonize and proliferate within EVTs. In a recent preprint, the Mysorekar group demonstrates the identification of a specific bacterial species, *Ralstonia insidiosa*, as a resident at the maternal-fetal interface [36]. The presence of *R. insidiosa* in decidual samples was demonstrated using species-specific FISH, and it was determined that bacteria were localized within extravillous trophoblasts. Seferovic et al. [37] further found evidence of bacteria in healthy term placentas using FISH and Warthin-Starry stains. This study also used contaminant controls and found that these controls had a different taxonomic makeup than samples, indicating that the bacteria found in the samples were not due to contamination. Younge et al. [31] recently collected placental, uterine, vaginal, rectal, amniotic fluid, and meconium samples from both term and preterm mother-infant pairs (delivered via cesarean section) and analyzed them using 16S rRNA gene amplicon sequencing and found significant similarities between the placental/uterine microbiome and the meconium and vaginal microbiomes. The authors also analyzed mouse fetal intestinal samples and confirmed the presence of a low number of bacteria in the fetal mouse gut.

In sum, there is a growing body of literature showing morphological evidence of bacteria in the fetal gut and at the maternal-fetal interface, particularly in the decidua.

## Group therapy: showing how each partner’s position makes sense and going forward together

With the papers highlighted herein and others, the pattern that appears is that early studies investigating the ‘placental microbiome’ were more focused on demonstrating an effect on clinical conditions and outcomes and considered several elements to demonstrate that the placental signatures were unique. Some papers which found significant differences between placental samples and negative controls highlighted dissimilarities between the proposed composition of the placental microbiome and potential sources of contamination, such as the vaginal/skin microbiomes [1, 4, 24] as evidence against the idea that the bacteria detected are due to contamination. However, these papers did not compare placental samples to vaginal/stool/skin samples from the same individual and acknowledged similarities to potential skin/vaginal contaminants in samples taken from the placenta or amniotic fluid [4, 6, 25, 27, 37]. Other papers pointed out the presence of bacteria in samples delivered via cesarean section [26], a lack of differences between cesarean and vaginally delivered samples [2, 25], or helped rule out the possibility of contamination during vaginal birth as a source of detected bacteria. However, the viability of the analytical tools used themselves in distinguishing genuine microbial signals from background noise was perhaps not as rigorously considered at first. Metanalysis looking at a variety of papers investigating the existence of a placental microbiome found that methodology varied considerably between studies, especially when looking at storage conditions for samples, method of DNA extraction, and method of analysis [38]. This pushed the groups who painstakingly identified kitomes, splash-omes, and risk of microbial DNA presence in the reagents themselves to suggest that *all* bacterial signal in the placenta or fetal gut/meconium *must* be contaminated [39]. However, the studies published thus far do not address whether and how absolute ‘sterility’ may be specifically maintained in this niche. We do not fully understand how the fetus emerges from a ‘sterile’ niche to face millions of bacteria in the birth canal and not be immunologically stunned. The placental barrier functions to eliminate most pathogens that enter the maternal-fetal interface using multiple mechanisms [39, 40]. We have not evaluated yet how this space is restricted to non-pathogenic bacteria. Are similar mechanisms employed to eliminate any non-pathogens that might enter this space?

**Fig. 2:**
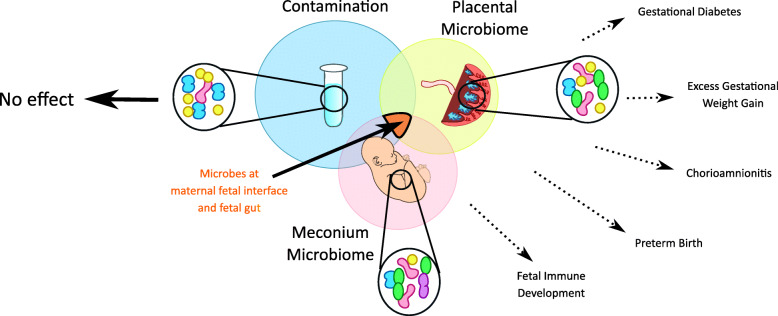
Betwixt the two stances of its ‘contamination’ and its a ‘microbiome’ lies a common overlap focused on specific microbes at the maternal fetal interface

Overall, the studies together have had the positive impact in that the number of controls used for methods of extraction, sampling location, and depth of analyses continues to become increasingly rigorous and thoughtful in *all* studies. A number of guidelines are emerging for ensuring rigor and reproducibility in microbiome/microbiota/localization analyses [18, 20, 36, 41–44]. The other common features that emerge are while it would appear based on the issue of signal versus noise that there does not appear to be a *bona fide* microbiome as defined as a collection of microbial communities in placental villi/parenchyma, samples which included maternal decidua and meconium find higher bacterial counts suggesting that these are niches to focus on. Furthermore, as highlighted above, there are a small number of bacteria found with visualization and localization approaches at both of these sites. These results suggest the presence of a low biomass, bacterial presence in healthy decidual/placental space and in the fetal gut and detailed analysis of bacterial location and shape and size appears to be key (Fig. 2). Who are these microbes, where they come from, when they appear during gestation, whether they are viable and/or actively replicating and what is their function, are all extraordinarily exciting and important avenues of investigation. It has been argued that normal bacterial presence can be beneficial for fetal tolerance and fetal preparation for post-natal exposure to a microbially dense world and inducing endotoxin tolerance. Recent reports have suggested that the fetus is exposed to a broad repertoire of antigens. Further, the fetal gut contains regulatory T cells and PLZF^+^ CD161^+^ memory T cells which are activated in response to bacterial , self-, and maternal antigens [45–47] in utero. Maternal and fetal commensal bacterial colonization could be playing a role in modulating fetal immune education and protective immunity prior to delivery. New studies are opening exciting new frontiers by identifying microbial components and metabolites, which may be the messengers that are vertically transmitted to the fetus during pregnancy to prime the developing fetal immune system [48, 49]. This would support Medawar’s concept that ‘actively acquired immunologic tolerance’ could occur as a result of in utero fetal exposure to microbial antigens transmitted vertically irrespective of the location of the microbial source [46, 50]. In conclusion, we set forth that rigorous methodologies, niche-specific analyses, and metabolite analysis, coupled with visualization and localization of bacteria, will allow us together to move forward and focus on the biological and functional implications of the presence/absence of microbes in the maternal-fetal interface and the developing fetus.

## Data Availability

Data sharing not applicable to this article as no datasets were generated or analyzed during the current study.
